# Inhibition of Rabies Virus by 1,2,3,4,6-Penta-*O*-galloyl-β-d-Glucose Involves mTOR-Dependent Autophagy

**DOI:** 10.3390/v10040201

**Published:** 2018-04-17

**Authors:** Zhongzhong Tu, Wenjie Gong, Yan Zhang, Ye Feng, Yan Liu, Changchun Tu

**Affiliations:** Key Laboratory of Jilin Province for Zoonosis Prevention and Control, Institute of Military Veterinary Medicine, Academy of Military Medical Sciences, Jilin 130122, China; tudong890901@163.com (Z.T.); gwj020406@163.com (W.G.); zhangyan.jl@163.com (Y.Z.); fengye621@126.com (Y.F.)

**Keywords:** PGG, IPS, RABV, antiviral, CVS-11

## Abstract

The compound 1,2,3,4,6-penta-*O*-galloyl-β-d-glucose (PGG), a gallotannin present in various plants such as *Rhus chinensis* Mill and *Paeonia suffruticosa*, has a broad spectrum of antiviral effects. The present study investigated its potency against infection of mice with rabies virus (RABV). Results demonstrated that PGG strongly inhibited virus titers (50-fold), viral mRNA expression (up to 90%), and protein synthesis in vitro. Importantly, we found that PGG not only suppressed viral adsorption and entry, but also directly inactivated RABV through suppression of autophagy by mediating activation of the mTOR-dependent autophagy signaling pathway. In vivo, PGG (10 mg/kg) alleviated the clinical symptoms and reduced the mortality of infected mice by 27.3%. Collectively, our results indicate that PGG has potent anti-RABV effect, and merits further investigation as an anti-RABV drug.

## 1. Introduction

Rsbies virus (RABV) is an enveloped, single-stranded negative-sense RNA virus belonging to the genus *Lyssavirus* of the Rhabdoviridae family, which invades the central nervous system and causes fatal encephalitis in mammals and humans. It has been estimated that over 60,000 humans die of rabies with more than 15 million humans worldwide receiving postexposure prophylaxis every year [1]. Rabies is still an incurable disease and practically the only way to prevent it is pre- or postexposure vaccination. Although several humans have reportedly survived rabies infection following treatment based on the “Milwaukee protocol” [2,3], this has been accompanied by severe neurological sequelae and the protocol has failed in most cases [4]. Treatment of rabies remains a major challenge, therefore, and requires the development of alternative strategies.

RABV involves five structural proteins: nucleoprotein (N), phosphoprotein (P), matrix protein (M), glycoprotein (G), and RNA-dependent RNA polymerase (L). Among them, P, as a constituent of the viral ribonucleoprotein complex, can not only interact with N and L, but also interact with several host proteins, such as dynein dynein light chain 8 (LC8), implicated in retrograde transport [5], and signal transducer and activator of transcriptions, implicated with interferon antagonism [6], which strongly suggests that P plays an important role in the life cycle of RABV [7].

The chemical compound 1,2,3,4,6-penta-*O*-galloyl-β-d-glucose (PGG) is a hydrolysable phenolic tannin present in many traditional medicinal herbs such as *Rhus chinensis* Mill and *Paeonia suffruticosa* [8,9]. PGG belongs to the group of gallotannins which also participates in the formation of ellagitannins. It has several biological activities, including antimutagenic [10], anti-inflammatory [11], anticancer [12,13] and antioxidant [14]. In addition, PGG has been reported to inhibit the replication of both DNA viruses, such as hepatitis B virus (HBV) [15] and herpes simplex virus (HSV) [9,16,17], and a broad range of RNA viruses, including human immunodeficiency virus (HIV-1) [18], respiratory syncytial virus (RSV) [19], hepatitis C virus (HCV) [20], human rhinoviruses (HRVs) [21], and influenza A virus (IAV) [22,23,24].

PGG has been reported to exert its antiviral effect by multiple mechanisms, including inhibition of the integrase and reverse transcriptase of HIV-1 [18] and the NS3 protease of HCV [20]. Moreover, PGG has been reported to inhibit the mTOR signaling pathway to enhance the scavenging effect of autolysosomes during HSV-1 infection, and to enhance autophagy resulting in the inhibition of influenza A viral growth and viral protein synthesis via modulation of Hsp90 induction and the mTOR/p70S6K signaling pathway [23]. However, the underlying mechanisms of the anti-RABV activity of PGG remained to be elucidated.

Autophagy is an evolutionarily conserved intracellular process which involves the formation of a double autophagosomal membrane, and plays an important role in the maintenance of cellular homeostasis and survival under stress conditions [25]. Three major pathways regulate autophagy induction: the mechanistic target of rapamycin (mTOR), which negatively regulates autophagy [26]; Atg6/Beclin-1 and binding of Beclin-1 by the antiapoptotic protein Bcl-2 [27]; and two ubiquitin-like conjugated processes associated with autophagosome formation [28]. Autophagy is an innate defense mechanism associated with the elimination of intracellular pathogens, via a process known as xenophagy, by degrading pathogens in autolysosomes [9,16,17]. PGG has been reported to exert its anti-HSV-1 and -IAV effects in association with autophagy [16,23]. Several studies have shown that RABV infection can induce autophagy in neuroblastoma cells and cellular autophagy benefits RABV replication [29,30]. However, whether PGG utilizes autophagy to inhibit the replication of RABV had not previously been investigated.

In the present study, the antiviral effect of PGG against RABV in vitro and in vivo was investigated, and its mechanism using mTOR-dependent autophagy to inhibit RABV replication was identified. As a comparison, we also included isoprinosine (IPS), an antiviral compound previously reported to inhibit RABV [31,32]. Our results provide strong evidence showing that PGG has the potential to be used as an effective inhibitor of RABV replication.

## 2. Materials and Methods

### 2.1. Compounds

**Figure 1:**
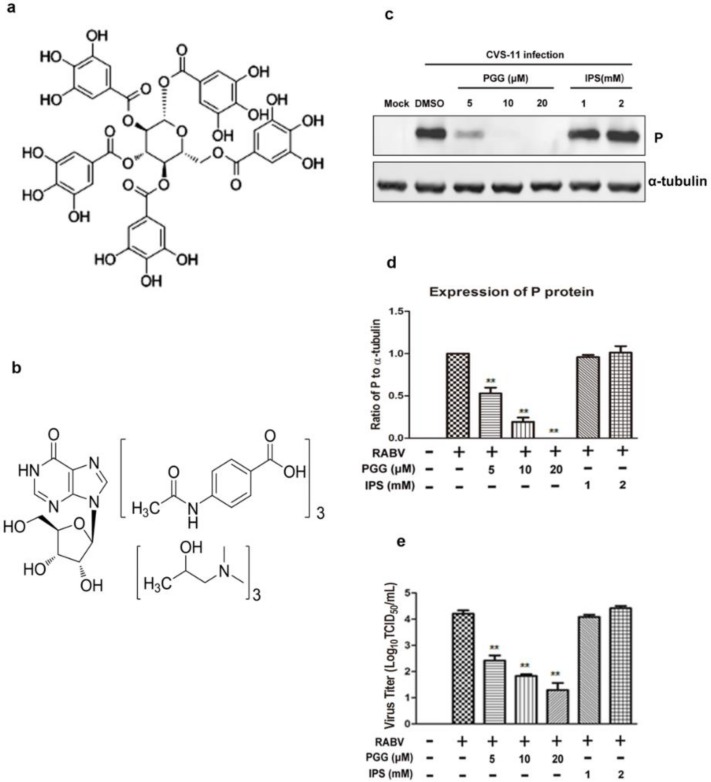
PGG directly inactivates rabies virus (RABV). Chemical structure of (**a**) 1,2,3,4,6-penta-*O*-galloyl-β-d-glucopyranose (PGG) and (**b**) isoprinosine (IPS). After incubation with PGG and IPS, RABV was inoculated with BHK-21 cells for 48 h, and (**c**) the expression level of viral P was analyzed by Western blotting. (**d**) The relative expression levels of viral P, normalizing to that of α-tubulin. (**e**) The virus titers of culture supernatants were determined by TCID_50_ assay and plotted. (** *p* < 0.01).

1,2,3,4,6-penta-*O*-galloyl-β-d-glucose (PGG, >98% purity, Figure 1a) and isoprinosine (IPS, Figure 1b) were purchased from Medchem Express (New Jersey, NJ, USA) and Toronto Research Chemicals (North York, TO, Canada). The compounds were dissolved in dimethyl sulfoxide (DMSO) and diluted in culture medium to a final concentration not exceeding 0.1% (*v*/*v*) to avoid toxicity.

### 2.2. Cell Cultures, Virus and Animals

BHK-21 cells were propagated in Minimum Essential Medium (MEM; Corning Inc., Corning, NY, USA) supplemented with 5% fetal bovine serum (FBS; Corning), 100 U/mL penicillin G, and 100 µg/mL streptomycin. RABV challenge virus standard (CVS)-11 strain was propagated in BHK-21 cells and stored at −80 °C. Virus titers were determined as 50% tissue culture infective doses (TCID_50_)/mL.

Kunming mice (female, 18–20 g) were purchased from the Changchun Institute of Biological Products. Sample collection and experimental infection of mice were reviewed and approved by the Administrative Committee on Animal Welfare of the Institute of Military Veterinary, Academy of Military Medical Sciences, China (Laboratory Animal Care and Use Committee Authorization, permit number: JSY-DW-2016-02). All animals were treated strictly in accordance to the Principles and Guidelines for Laboratory Animal Medicine (2006) of the Ministry of Science and Technology, China.

### 2.3. Cytotoxic Assay

Plastic 96-well plates were seeded with BHK-21 cells and incubated at 37 °C for 24 h. The culture medium was replaced with fresh medium containing various concentrations of PGG (2.5–100 μM) or IPS (0.25–10 mM) and incubated for 48 h. The surviving cell fraction was determined using the sulforhodamine B (SRB) assay as described previously [33,34]. Nonlinear regression analysis of the dose–response curves was used to determine 50% cytotoxic concentrations (CC_50_).

### 2.4. Antiviral Assay

Twelve-well plates were seeded with BHK-21 cells and incubated as described above, then infected with CVS-11 (MOI = 0.1) at 37 °C for 1 h. After washing with PBS, the fresh culture medium was added to the cells containing various concentrations of PGG (1.25–20 μM) or IPS (0.125–2 mM) and incubated for 48 h. The antiviral effects were determined by calculating the reduction of virus as TCID_50_ [35]. The inhibition rate (%) = [(control virus titer − treated virus titer)/(control virus titer)] × 100. The 50% inhibitory concentration (IC_50_) was calculated by regression analysis of the dose–response curves. The results were expressed using the selectivity index (SI = CC_50_/IC_50_).

### 2.5. Virus Titration

Ninety-six-well plates were seeded with BHK-21 cells and incubated as described above, then infected with 10-fold serial dilutions of the virus. After incubation for 72 h, virus titers were determined by a direct fluorescent antibody (DFA) assay [36]. Cells showing specific apple-green fluorescence were counted as being RABV-infected and virus titers were calculated as TCID_50_/mL by the Spearman–Kärber formula [37].

### 2.6. Time-of-Addition Assay

Twelve-well plates were seeded with BHK-21 cells and cultured overnight. 10 μM PGG was added at various stages of the virus infection [38]: 1 or 2 h before RABV infection (−2, −1 h p.i.), together with RABV infection (0 h p.i.), and at 1–24 h post-RABV infection (1–24 h p.i.). After incubation for 48 h postinfection (p.i.), the culture supernatants were collected to determine virus titers as described above. Cell monolayers were lysed by radio-immunoprecipitation assay (RIPA) buffer and the viral P protein in infected cells were analysed by Western blotting.

### 2.7. Viral Adsorption, Entry and Inactivation Assay

Twelve-well plates were seeded with BHK-21 cells and cultured overnight. Cells were pretreated with various concentrations of PGG before being infected with CVS-11 (MOI = 0.1) at 4 °C for 1 h, and viral adsorption was assayed according to the method of Yang et al. [39]. There were two duplicate plates: a part of the cells was immediately lysed for qRT-PCR detection of the RABV P gene after viral infection; the other equal part was incubated for another 48 h. The virus titers in the supernatant and P protein expression in cells were detected respectively as described above.

BHK-21 cells were prepared as above and infected with CVS-11 at 4 °C for 1 h, for assay of viral entry according to the method of Luo et al. [40]. There were two duplicate plates: a part of the cells was immediately lysed for qRT-PCR detection of the RABV P gene after PGG treatment; the other equal part was incubated for another 48 h. The virus titers in the supernatant and P protein expression in cells were detected respectively as described above. 

CVS-11 was coincubated with various concentrations of PGG at 37 °C for 1 h, and inactivation of RABV was assayed according to the method of Isaacs et al. [41]. After incubation for 48 h p.i., the virus titers in the supernatant were titrated and the viral P protein in the cell sheet was detected as described above.

### 2.8. Quantitative Real-Time Reverse Transcription PCR (qRT-PCR)

Twelve-well plates were seeded with BHK-21 cells, cultivated, and infected with CVS-11 as described above. The culture medium was replaced with fresh medium containing various concentrations of PGG and incubated for 48 h. Viral genome copies were quantified by qRT-PCR using the Agilent-Stratagene Mx3000P Q-PCR System according to a previously described protocol [42]. The primer sequences for RABV P amplification were as follows: forward: 5′-CCTCCTTTCAAACCATCCCA-3′ and reverse: 5′-ACTTGCCTTCTCCCACCCTA-3′. The primer sequences for GAPDH (internal control) were as follows: forward: 5′-GTTCAAAGGCACAGTCAAGG-3′ and reverse: 5′-ACGCCAGTAGACTCCACAAC-3′. PCR conditions were as follows: denaturation at 95 °C for 30 s, followed by 40 cycles of 95 °C for 5 s and 60 °C for 1 min. Melting curves were performed to verify the specificity of products.

### 2.9. In-Cell Western Assay

Ninety-six-well plates were seeded with BHK-21 cells, infected with CVS-11, and treated with PGG as described above, and mock-infected was the negative control. After incubation for 48 h, cells were fixed with 4% paraformaldehyde for 30 min at room temperature, blocked with Odyssey blocking buffer (LI-COR) at 37 °C for 1 h, then incubated with mouse polyclonal antibody against RABV P protein (prepared by our laboratory) at 37 °C for 1 h. After washing with PBST, cells were incubated with Alexa Fluor 488 donkey antimouse IgG (H + L) at 37 °C for 1 h. After washing with PBST, the plates were observed using an Odyssey infrared imaging system (LI-COR).

### 2.10. Detection of mTOR-Dependent Autophagy by Western Blotting

Six-well plates were seeded with BHK-21 cells and cultured overnight before treatment with rapamycin or MHY1485 in MEM medium without FBS for 1 h. Following infection with CVS-11 for 1 h, cells were cultured in medium containing PGG, rapamycin, or MHY1485 for a further 24 h, then lysed in RIPA buffer containing Halt Protease Inhibitor Cocktail (Thermo, Waltham, MA, USA). Cell lysates (40 μg) were then subjected to SDS-PAGE. Western blotting was conducted with the indicated antibody according to a previously described protocol [43]. Protein bands were detected by an Odyssey infrared imaging system and the bands were quantified by ImageJ software (version 1.36b, NIH, Bethesda, MD, USA).

### 2.11. In Vivo Assay of PGG Anti-RABV Effects

Animal experiments were performed according to protocols approved by the Institutional Animal Care and Use Committees of the Academy of Military Medical Sciences (Laboratory Animal Care and Use Committee Authorization, permit number: JSY-DW-2016-02, in September 2015). Kunming mice were randomly divided into 10 groups (*n* = 10 or 11). Five groups were injected intramuscularly (IM) in both hind legs with a lethal dose of CVS-11 (10 LD_50_, 10^6.5^ TCID_50_) and 1 h later with intraperitoneal (IP) injections of saline or various concentrations of PGG or IPS daily for three consecutive days. The other five groups were used as healthy controls and treated identically, but without virus. All animals were observed daily for 21 days, and clinical signs were scored as described previously [44].

### 2.12. Statistical Analysis

The data are expressed as the mean ± standard error of mean (SEM), and statistical evaluation was performed using one-way analysis of variance (ANOVA). Statistical significance was analyzed by the Student’s *t*-test and the chi-square test using GraphPad Prism software (version 5.0, La Jolla, CA, USA), and *p* < 0.05 was considered as being statistically significant.

## 3. Results

### 3.1. Cytotoxicity and Inactivation of RABV by PGG

The cytotoxicity assay showed that the CC_50_ of PGG and IPS was 22.48 ± 2.20 μM and 1.946 ± 0.180 mM, respectively (Table 1), with no apparent cytotoxicity observed at working concentrations of up to 10 μM (PGG) and 1 mM (IPS).

The inactivation assay showed that the virus titers in PGG-treated groups were decreased 65–625-fold and viral P expression was also reduced, whereas there were no significant differences in titers and P protein expression in IPS-treated groups as compared with controls (Figure 1c–e).

### 3.2. Antiviral Effects of PGG In Vitro

As determined by antiviral assay, the IC_50_ and selectivity indices (SI = CC_50_/IC_50_) of PGG and IPS were 3.90 ± 0.82 μΜ and 5.76, and 0.3327 ± 0.0364 mM and 5.85, respectively (Table 1), showing that both compounds had significant anti-RABV effects in vitro.

**Figure 2:**
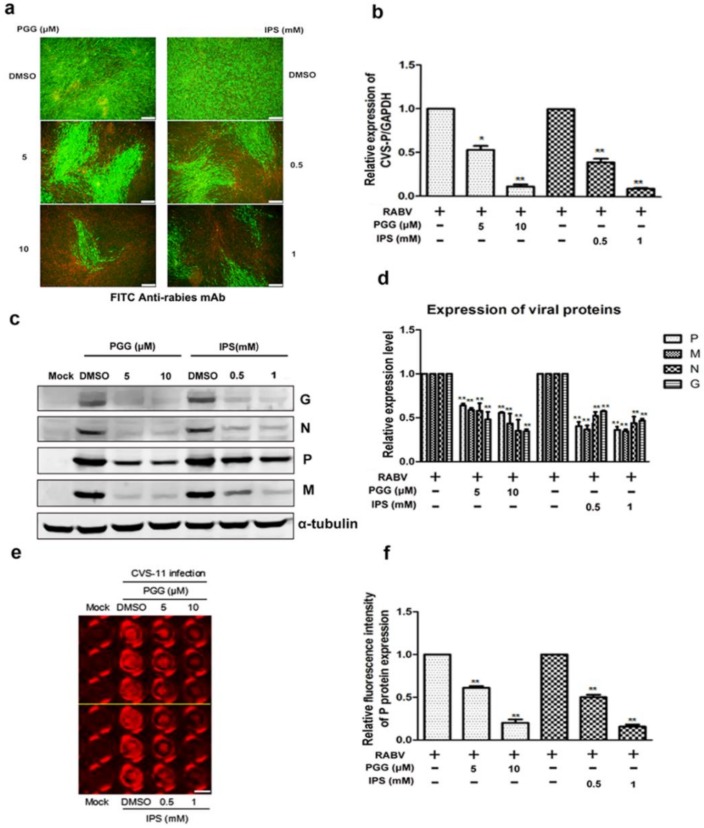
PGG inhibits the replication of RABV. Infected BHK-21 cells were treated with PGG or IPS for 48 h, and (**a**) the viral particles were detected by direct fluorescent antibody (DFA) assay; apple-green fluorescence represents fluorescein isothiocyanate (FITC)-labeled RABV, scale bar = 100 μm (**b**) The viral P gene mRNA expression level was detected by qRT-PCR. (**c**) The viral proteins’ expression levels were analyzed by Western blotting. (**d**) The relative expression levels of viral P, M, N, and G, normalizing to that of α-tubulin. (**e**) The viral P protein expression level was detected by in-cell Western assay; red fluorescence represents RABV P protein, scale bar = 100 μm (**f**) The relative expression levels of viral P, normalizing to that of the control. (* *p* < 0.05; ** *p* < 0.01).

As shown in Figure 2a, both PGG and IPS significantly inhibited the virus growth at 48 h p.i. Within the same time period, RNA copies of the P gene were decreased by 50–90% (PGG) and 60–93% (IPS) and the expression of CVS-11 G, N, P, and M proteins (Preparation of anti-RABV P, G, M and N polyclonal antibodies were listed in Supplementary Figures S1 and S2) were inhibited by PGG and IPS (Figure 2b–d). Moreover, the P protein level was detected by incubating anti-RABV P protein polyclonal antibody and Alexa Fluor 488 donkey anti-mouse IgG (H + L) in the in-cell Western assay, and the result showed that the red fluorescence intensity of P protein was significantly decreased by 42–80% and 50–84% by PGG and IPS, respectively, as compared with controls (Figure 2e,f).

**Figure 3:**
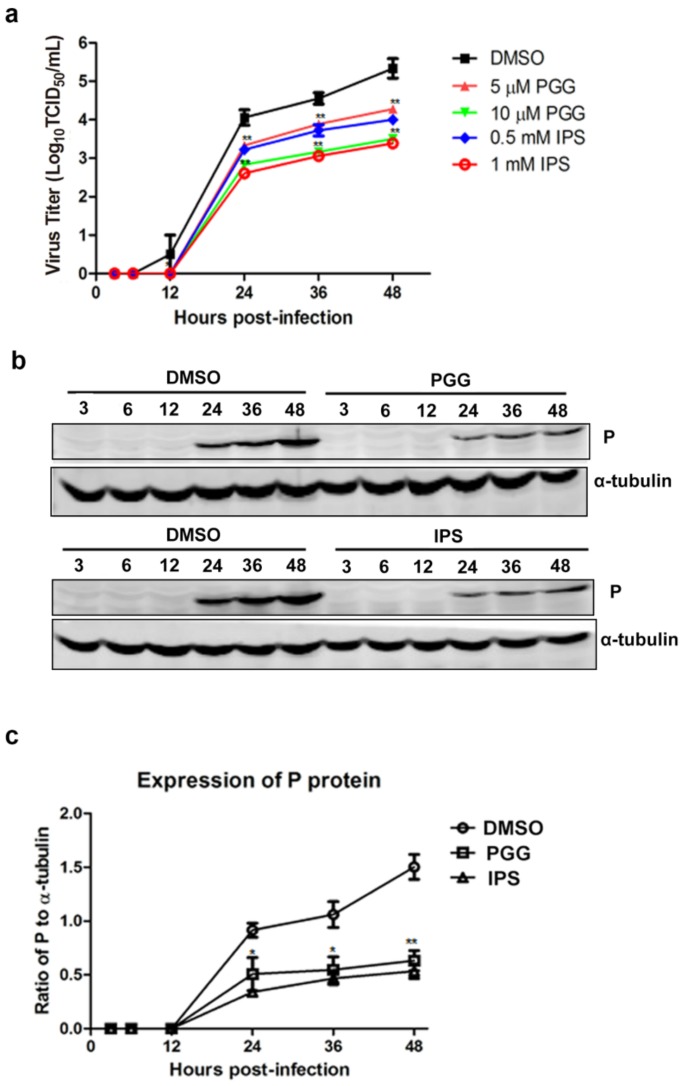
PGG inhibits viral yields and viral protein synthesis. Infected BHK-21 cells were treated with PGG or IPS at various time points. (**a**) Growth kinetics of infected BHK-21 cells during a 48-h period p.i. after treatment with PGG or IPS; (**b**) The viral P protein expression level was analyzed by Western blotting during a 48-h period p.i.; (**c**) The relative expression levels of viral P, normalizing to that of α-tubulin. (* *p* < 0.05; ** *p* < 0.01).

As shown in Figure 3a, virus titers were decreased by approximately 11–50-fold and 11–60-fold by PGG and IPS treatment, respectively, between 12 h and 48 h, as compared with controls. Furthermore, viral P protein levels were reduced at all time points (Figure 3b,c). Taken altogether, the above results show that PGG significantly inhibits both the gene replication and protein synthesis of RABV with an efficacy similar to that of IPS.

### 3.3. PGG Inhibits the Adsorption and Entry of RABV

**Figure 4:**
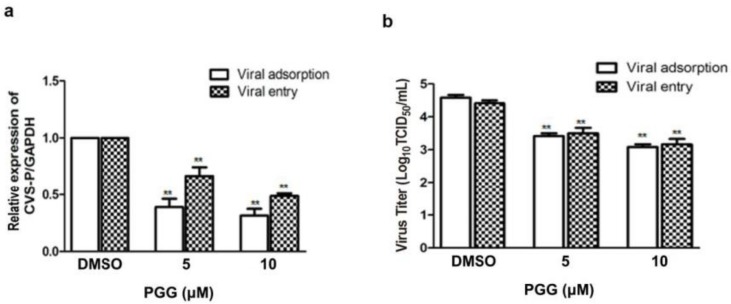

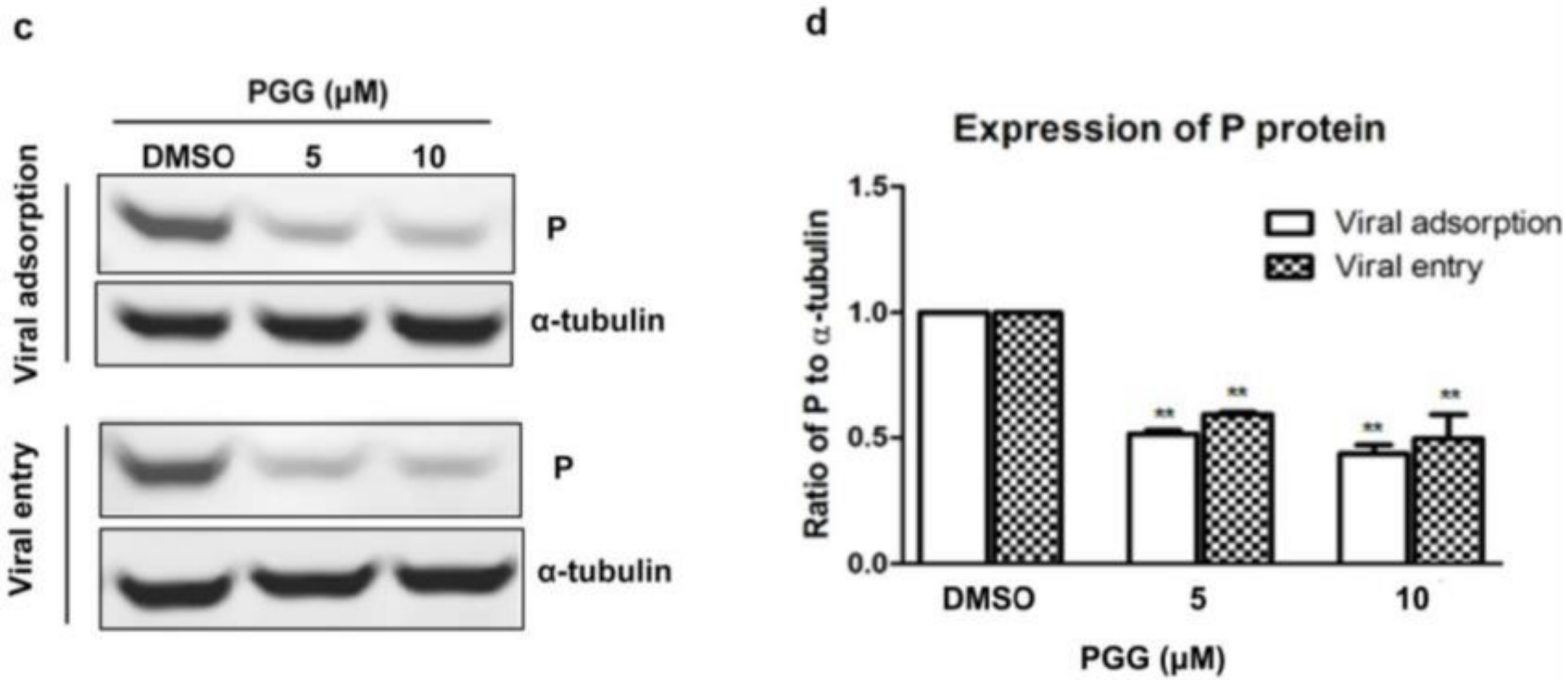
PGG inhibits RABV adsorption and entry. For viral adsorption or entry assay, BHK-21 cells were treated with PGG for 1 h. (**a**) The vira1 P gene mRNA expression level was detected by qRT-PCR; (**b**) The virus titers of culture supernatants were determined by TCID_50_ assay and plotted; (**c**) The expression level of viral P protein was analyzed by Western blotting; (**d**) The relative expression levels of viral P, normalizing to that of α-tubulin. (** *p* < 0.01).

Viral adsorption and entry was assayed after treatment with PGG. Results showed that PGG (5 or 10 μΜ) decreased viral P gene copies to 60% or 70%, respectively, at the viral adsorption stage and 35% or 53%, respectively, at the viral entry stage, as compared with controls (Figure 4a). After incubation for 48 h postadsorption, the virus titers were decreased by 15- and 32-fold by PGG (5 and 10 μΜ, respectively), and for entry, by 10- and 21-fold (Figure 4b), consistent with the decreased RABV P protein expression (Figure 4c,d).

### 3.4. Time Course of PGG-Mediated Inhibition of RABV Replication

**Figure 5:**
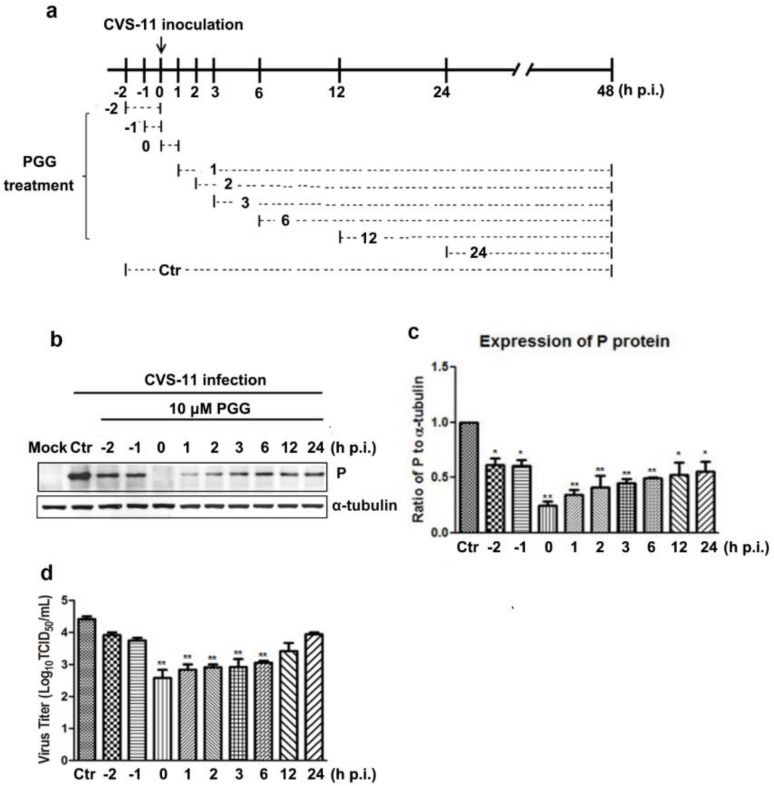
Time-of-addition assay. (**a**) Schematic representation of the experimental design. BHK-21 cells were treated with 10 μM PGG at the indicated times. (**b**) The viral P protein expression level was analyzed by Western blotting at 48 h p.i. (**c**) The relative expression levels of viral P, normalizing to that of α-tubulin. (**d**) The virus titers of culture supernatants were determined by TCID_50_ assay and plotted. Ctr is the abbreviation of control. (* *p* < 0.05; ** *p* < 0.01).

Virus P protein levels and virus titers were determined at different infection times following PGG treatment (Figure 5a). Results showed that PGG significantly inhibited RABV growth, as evidenced by decreased P protein levels and virus titers (5–100-fold) (Figure 5b–d). These results indicate that PGG inhibits RABV replication mainly at early stage of infection.

### 3.5. PGG Inhibits RABV Replication Via Suppressing mTOR-Dependent Autophagy

**Figure 6:**
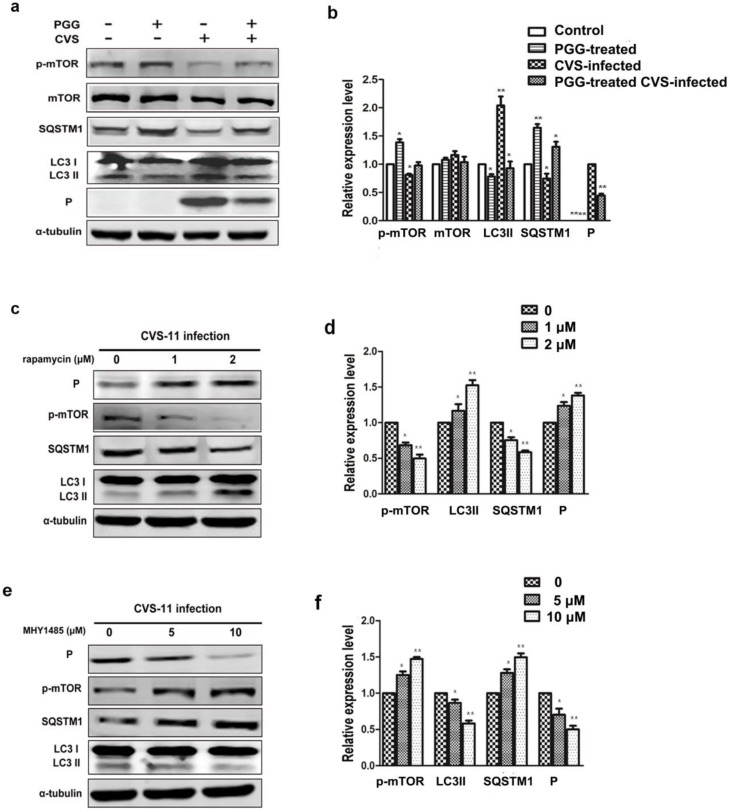
PGG inhibits the replication of RABV via mTOR-associated autophagy. (**a**) mTOR pathway-associated proteins and autophagy markers were analyzed by Western blotting, after CVS-infected BHK-21 cells treated with PGG for 24 h. (**b**) The relative expression levels of mTOR, p-mTOR, SQSTM1, LC3, and viral P protein, normalizing to that of α-tubulin. BHK-21 cells were pretreated with rapamycin (1 or 2 μM) or MHY1485 (5 or 10 μM) for 1 h, then infected with CVS-11 and cultured in medium containing rapamycin or MHY1485 for 24 h. (**c**, **e**) p-mTOR, autophagy markers, and P protein were analyzed by Western blotting. (**d**, **f**) The relative expression levels of p-mTOR, SQSTM1, LC3, and viral P, normalizing to that of α-tubulin. (* *p* < 0.05; ** *p* < 0.01).

We investigated whether PGG inhibited RABV replication by suppressing RABV-induced autophagy, and if so, by which pathway. Results showed that the autophagy marker protein LC3-II was decreased and the autophagic flux-specific protein SQSTM1 was increased (Figure 6a,b) after treatment with PGG, and furthermore, phosphorylation of mTOR was significantly increased. To further confirm that PGG-induced inhibition of autophagy is mTOR pathway-dependent, BHK-21 cells were pretreated with the mTOR inhibitor rapamycin or activator MHY1485 before infection with CVS-11. As shown in Figure 6c–f, rapamycin inhibited the activation of mTOR, resulting in the increase of both cellular autophagy and P protein levels. In contrast, MHY1485 promoted the activation of mTOR, resulting in suppression of both cellular autophagy and P protein levels.

**Figure 7:**
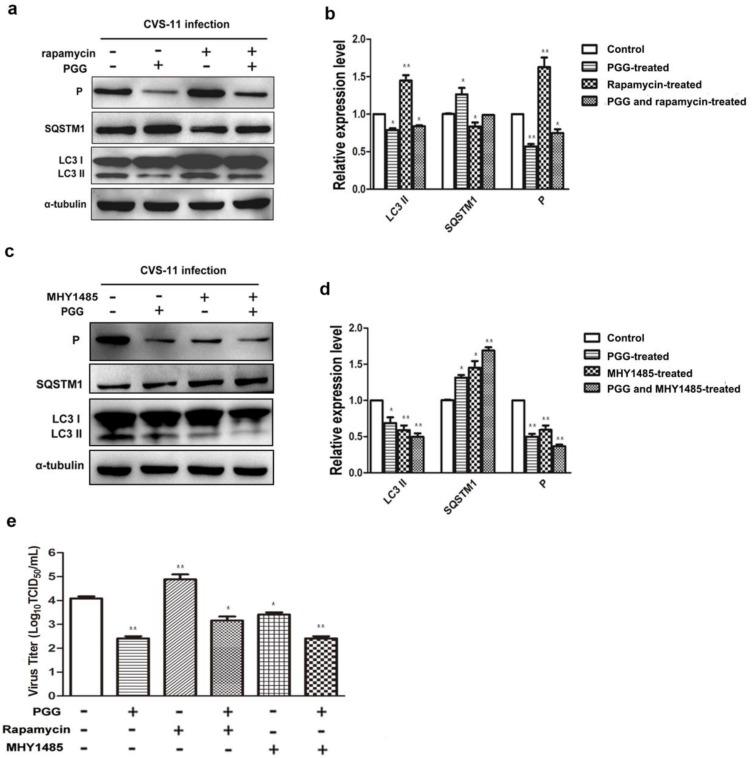
PGG inhibits the replication of RABV via suppression of mTOR-dependent autophagy. BHK-21 cells were pretreated with rapamycin (1 μM) or MHY1485 (5 μM) for 1 h, then infected with CVS-11 and PGG, rapamycin or MHY1485 respectively or in combination for 24 h. (**a**, **c**) autophagy markers and P protein were analyzed by Western blotting. (**b**, **d**) The relative expression levels of SQSTM1, LC3 and viral P, normalizing to that of α-tubulin. (**e**) The virus titers of culture supernatants were determined by TCID_50_ assay and plotted. (* *p* < 0.05; ** *p* < 0.01).

Importantly, we found that the inhibitory effect of PGG was diminished when cells were pretreated with rapamycin; meanwhile, the inhibitory effect of PGG was enhanced when cells were pretreated with MHY1485, which strongly supports the conclusion that mTOR plays a key role in PGG-induced autophagy inhibition (Figure 7a–e).

### 3.6. Antiviral Effects of PGG In Vivo

**Figure 8:**
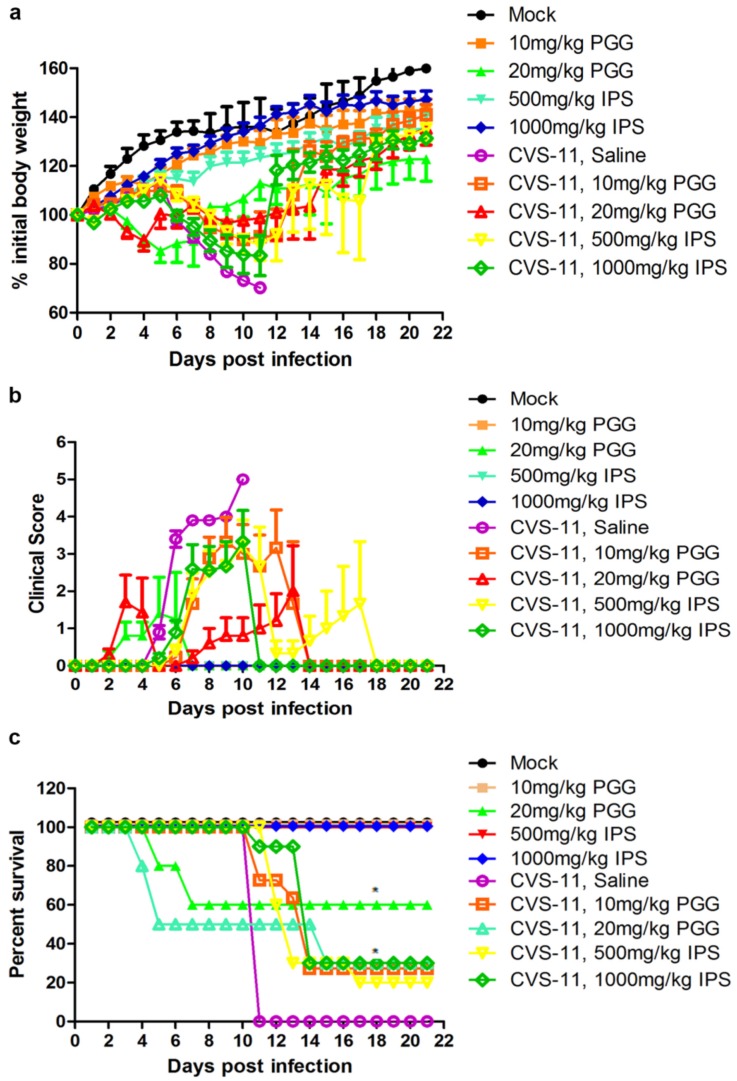
PGG alleviates the clinical symptoms and reduces the mortality of RABV-infected mice. Kunming mice (*n* = 10 or 11) were challenged intramuscularly (IM) with 10 LD_50_ of lethal CVS-11 and treated with PGG or IPS (indicated concentration) by intraperitioneal (IP) injection 1 h p.i. and given daily by IP injection for 3 day, and observed for body weight change (**a**); clinical signs (**b**); and survival (**c**) for 21 day. (* *p* < 0.05; ).

Kunming mice were infected or uninfected with CVS-11 and 1 h later with IP injection of PGG (10 or 20 mg/kg) for three days. An identical group was treated with IPS groups (500 or 1000 mg/kg) in place of PGG. Body weights, clinical signs, and mortalities were monitored until 21 days p.i. As shown in Figure 8, overall clinical signs and body weight loss were alleviated in all challenged mice, no matter whether they received PGG or IPS treatment, and gradual recovery was observed after 11–14 days post challenge, as compared with untreated challenged controls, which all developed incoordination at 4–6 days p.i. and rapidly progressed to hunching, trembling, paralysis, and finally 100% mortality. It is noted that 27.3% of challenged mice survived in the 10 mg/kg PGG group, while 30% of challenged mice survived in the 20 mg/kg PGG group, but this PGG dose showed toxicity and caused 40% mortality (4/10) of unchallenged mice. For the IPS groups, 20% (500 mg/kg) and 30% (1000 mg/kg) of challenged mice survived and showed no obvious toxicity (Figure 8c).

## 4. Discussion

Several phenolic compounds and their derivatives have been reported to inhibit RABV in vitro, such as catechin, quercetin, 3,4,5-trimethoxy-acetophenone, and 3,4,5-trimethoxybenzoic acid ethyl ester [45]. PGG is a hydrolysable gallotannin of a phenolic compound with five free hydroxyl groups of glucose and gallic acid acylation (see Figure 1a). Interestingly, PGG has a symmetrical chemical structure, resulting in greater hydrophobicity and lower oxidative effect in an aqueous environment than epigallocatechin-3-gallate, epigallocatechin, and hexagalloylucose [46,47]. Several gallates possess anti-HSV and anticytomegalovirus activity due to inhibition of viral adsorption and entry by their hydroxyl groups [48]. The galloyl moieties of gallic acid and galloyl glucose also have anti-HIV-1 activity [49], suggesting that the gallate structure of PGG, not its gallic acid moieties, likely plays a critical role in the inhibition of RABV replication, and its five free hydroxyl groups might be involved in inhibition of RABV adsorption and entry.

Previous studies have reported that phenolic compounds exert their antiviral effects not only by directly inactivating the virus before cellular invasion [41,50], but also by suppression of the replication of the viral genome, synthesis of viral proteins, and production of progeny viruses after entrance of the virus into cells. Herein, we have shown that PGG exerts its anti-RABV effect by all these activities. To convincingly demonstrate the antiviral potency of PGG, we tested isoprinosine (IPS) in parallel to compare the efficacy and toxicity of these two compounds, since the latter has been reported to potently inhibit the growth of RABV ERA and VA319 strains in cell cultures [31]. Our results showed that PGG had an efficacy against rabies virus in vitro similar to that of IPS. Glasgow and Galasso reported that IPS (300 mg/kg) reduced the mortality of street RABV-infected mice by 16%, although this effect was not considered statistically significant [32]. The higher doses used in the present work (IPS: 500 and 1000 mg/kg and PGG: 10 and 20 mg/kg) were selected according to the published literature [46,47]. Both doses showed an inhibitory effect, protecting about 30% of challenged mice from death; however, the PGG dose of 20 mg/kg produced some toxicity in unchallenged mice, indicating that 10 mg/kg is the safe dose. All 10 untreated saline control animals died at 11 days p.i. Deaths in the three treated groups occurred between 12–14 days p.i., and all dead mice were shown to be positive for RABV by fluorescent antibody test (FAT), except for the 20 mg/kg PGG group, with 50% (5/10) of the challenged mice dying before five days p.i with negative FAT staining, indicating that these mice died of the toxicity of PGG. The results indicate that PGG exerted an anti-RABV effect in vivo similar to that of IPS, but 20 mg/kg PGG had a certain toxicity.

Autophagy has been found to play critical roles in facilitating or inhibiting viral replication in multiple cellular signaling pathways, such as Atg6/Beclin-1, Beclin-1/Bcl-2, MAPK/ERK1/2, and ULK1/Atg1/mTOR [29,51,52]. RABV infection has been reported to induce autophagy during viral replication in human and mouse neuroblastoma cell lines, via downregulating the CASP2/caspase 2 pathway or by activating AMPK–AKT–mTOR and AMPK–MAPK pathways. Additionally, the promotion of autophagy by rapamycin or Earle’s balanced salt solution enhanced RABV replication, while inhibition of autophagy by wortmannin or 3-methyladenine suppressed RABV replication [29,30]. Considering previous studies showing that PGG can inhibit the replication of HSV-1 and IAV by inducing autophagy, PGG could also induce autophagy in cancer cells [53,54]. Therefore, we further explored whether PGG exerts an anti-RABV effect through reversing RABV-induced autophagy, and which mechanism was involved in PGG-associated autophagy. Our results demonstrated that PGG significantly suppressed autophagy and inhibited the replication of RABV, in accompaniment with decreased LC3-II expression levels and increased phosphorylation of mTOR. Importantly, we found that the inhibition of mTOR diminished the anti-RABV efficacy of PGG, accompanied with decreased mTOR phosphorylation and increased LC3-II, as compared with PGG monotreatment, which strongly supports the conclusion that mTOR plays a key role in PGG-induced autophagy inhibition.

In summary, our study showed that PGG could inhibit RABV replication in cell cultures and demonstrated an antiviral effect in RABV-infected mice, indicating that this compound could be a potential candidate for the development of an anti-RABV drug [51].

## Figures and Tables

**Table 1:** Cytoxicity, anti-RABV effect, and selectivity indices of tested compounds.

Compounds	CC_50_ (95% CL)	IC_50_ (95% CL)	SI (CC_50_/IC_50_)
PGG (μM)	22.48 (20.327–24.921)	3.90 (3.145–4.837)	5.76
IPS (mM)	1.946 (1.766–2.145)	0.3327 (0.3000–0.3691)	5.85

CC50: 50% cytotoxic concentration; IC50: 50% inhibitory concentration; SI: selectivity index; CL: confidence level. PGG: 1,2,3,4,6-penta-*O*-galloyl-β-d-glucose; IPS: isoprinosine.

## Supplementary Materials

The following are available online at http://www.mdpi.com/1999-4915/10/4/201/s1, Figure S1: Preparation and identification of anti-RABV P protein polyclonal antibody, Figure S2: Preparation and identification of anti-RABV M, N, and G proteins polyclonal antibodies.

## References

[1] Singh R., Singh K.P., Cherian S., Saminathan M., Kapoor S., Manjunatha Reddy G.B., Panda S., Dhama K. (2017). Rabies—Epidemiology, pathogenesis, public health concerns and advances in diagnosis and control: A comprehensive review. Vet. Q..

[2] Willoughby R.E., Tieves K.S., Hoffman G.M., Ghanayem N.S., Amlie-Lefond C.M., Schwabe M.J., Chusid M.J., Rupprecht C.E. (2005). Survival after treatment of rabies with induction of coma. N. Engl. J. Med..

[3] Wilde H., Hemachudha T. (2015). The “Milwaukee protocol” for treatment of human rabies is no longer valid. Pediatr. Infect. Dis. J..

[4] Zeiler F.A., Jackson A.C. (2016). Critical Appraisal of the Milwaukee Protocol for Rabies: This Failed Approach Should Be Abandoned. Can. J. Neurol. Sci..

[5] Raux H., Flamand A., Blondel D. (2000). Interaction of the rabies virus P protein with the LC8 dynein light chain. J. Virol..

[6] Wiltzer L., Okada K., Yamaoka S., Larrous F., Kuusisto H.V., Sugiyama M., Blondel D., Bourhy H., Jans D.A., Ito N. (2014). Interaction of rabies virus P-protein with STAT proteins is critical to lethal rabies disease. J. Infect. Dis..

[7] Real E., Rain J.C., Battaglia V., Jallet C., Perrin P., Tordo N., Chrisment P., D’Alayer J., Legrain P., Jacob Y. (2004). Antiviral drug discovery strategy using combinatorial libraries of structurally constrained peptides. J. Virol..

[8] Kim Y.H., Yang X., Yamashita S., Kumazoe M., Huang Y., Nakahara K., Won Y.S., Murata M., Lin I.C., Tachibana H. (2015). 1,2,3,4,6-penta-*O*-galloyl-β-d-glucopyranose increases a population of T regulatory cells and inhibits IgE production in ovalbumin-sensitized mice. Int. Immunopharmacol..

[9] Pei Y., Xiang Y.F., Chen J.N., Lu C.H., Hao J., Du Q., Lai C.C., Qu C., Li S., Ju H.Q. (2011). Pentagalloylglucose downregulates cofilin1 and inhibits HSV-1 infection. Antiviral Res..

[10] Sakai Y., Nagase H., Ose Y., Kito H., Sato T., Kawai M., Mizuno M. (1990). Inhibitory action of peony root extract on the mutagenicity of benzo[a]pyrene. Mutat. Res..

[11] Kang D.G., Moon M.K., Choi D.H., Lee J.K., Kwon T.O., Lee H.S. (2005). Vasodilatory and anti-inflammatory effects of the 1,2,3,4,6-penta-*O*-galloyl-β-d-glucose (PGG) via a nitric oxide-cGMP pathway. Eur. J. Pharmacol..

[12] Mizushina Y., Zhang J., Pugliese A., Kim S.H., Lu J. (2010). Anti-cancer gallotannin penta-*O*-galloyl-β-d-glucose is a nanomolar inhibitor of select mammalian DNA polymerases. Biochem. Pharmacol..

[13] Zhang J., Li L., Kim S.H., Hagerman A.E., Lu J. (2009). Anti-cancer, anti-diabetic and other pharmacologic and biological activities of penta-galloyl-glucose. Pharm. Res..

[14] Abdelwahed A., Bouhlel I., Skandrani I., Valenti K., Kadri M., Guiraud P., Steiman R., Mariotte A.M., Ghedira K., Laporte F. (2007). Study of antimutagenic and antioxidant activities of gallic acid and 1,2,3,4,6-pentagalloylglucose from *Pistacia lentiscus*. Confirmation by microarray expression profiling. Chem. Biol. Interact..

[15] Lee S.J., Lee H.K., Jung M.K., Mar W. (2006). In vitro antiviral activity of 1,2,3,4,6-penta-*O*-galloyl-beta-d-glucose against hepatitis B virus. Biol. Pharm. Bull..

[16] Pei Y., Chen Z.P., Ju H.Q., Komatsu M., Ji Y.H., Liu G., Guo C.W., Zhang Y.J., Yang C.R., Wang Y.F. (2011). Autophagy is involved in anti-viral activity of pentagalloylglucose (PGG) against Herpes simplex virus type 1 infection in vitro. Biochem. Biophys. Res. Commun..

[17] Jin F., Ma K., Chen M., Zou M., Wu Y., Li F., Wang Y. (2016). Pentagalloylglucose Blocks the Nuclear Transport and the Process of Nucleocapsid Egress to Inhibit HSV-1 Infection. Jpn. J. Infect. Dis..

[18] Ahn M.J., Kim C.Y., Lee J.S., Kim T.G., Kim S.H., Lee C.K., Lee B.B., Shin C.G., Huh H., Kim J. (2002). Inhibition of HIV-1 integrase by galloyl glucoses from *Terminalia chebula* and flavonol glycoside gallates from *Euphorbia pekinensis*. Planta Med..

[19] Yeo S.J., Yun Y.J., Lyu M.A., Woo S.Y., Woo E.R., Kim S.J., Lee H.J., Park H.K., Kook Y.H. (2002). Respiratory syncytial virus infection induces matrix metalloproteinase-9 expression in epithelial cells. Arch. Virol..

[20] Duan D., Li Z., Luo H., Zhang W., Chen L., Xu X. (2004). Antiviral compounds from traditional Chinese medicines Galla Chinese as inhibitors of HCV NS3 protease. Bioorg. Med. Chem. Lett..

[21] Ngan L.T., Jang M.J., Kwon M.J., Ahn Y.J. (2015). Antiviral activity and possible mechanism of action of constituents identified in *Paeonia lactiflora* root toward human rhinoviruses. PLoS ONE.

[22] Liu G., Xiong S., Xiang Y.F., Guo C.W., Ge F., Yang C.R., Zhang Y.J., Wang Y.F., Kitazato K. (2011). Antiviral activity and possible mechanisms of action of pentagalloylglucose (PGG) against influenza A virus. Arch. Virol..

[23] Liu G., Zhong M., Guo C., Komatsu M., Xu J., Wang Y., Kitazato K. (2016). Autophagy is involved in regulating influenza A virus RNA and protein synthesis associated with both modulation of Hsp90 induction and mTOR/p70S6K signaling pathway. Int. J. Biochem. Cell Biol..

[24] Liu G., Xiang Y., Guo C., Pei Y., Wang Y., Kitazato K. (2014). Cofilin-1 is involved in regulation of actin reorganization during influenza A virus assembly and budding. Biochem. Biophys. Res. Commun..

[25] Levine B., Klionsky D.J. (2004). Development by self-digestion: Molecular mechanisms and biological functions of autophagy. Dev. Cell.

[26] Jung C.H., Ro S.H., Cao J., Otto N.M., Kim D.H. (2010). mTOR regulation of autophagy. FEBS Lett..

[27] Pattingre S., Tassa A., Qu X., Garuti R., Liang X.H., Mizushima N., Packer M., Schneider M.D., Levine B. (2005). Bcl-2 antiapoptotic proteins inhibit Beclin 1-dependent autophagy. Cell.

[28] Fujita N., Itoh T., Omori H., Fukuda M., Noda T., Yoshimori T. (2008). The Atg16L complex specifies the site of LC3 lipidation for membrane biogenesis in autophagy. Mol. Biol. Cell.

[29] Liu J., Wang H., Gu J., Deng T., Yuan Z., Hu B., Xu Y., Yan Y., Zan J., Liao M. (2017). BECN1-dependent CASP2 incomplete autophagy induction by binding to rabies virus phosphoprotein. Autophagy.

[30] Peng J., Zhu S., Hu L., Ye P., Wang Y., Tian Q., Mei M., Chen H., Guo X. (2016). Wild-type rabies virus induces autophagy in human and mouse neuroblastoma cell lines. Autophagy.

[31] Hernandez-Jauregui P., Gonzalez-Vega D., Cruz-Lavin E., Hernandez-Baumgarten E. (1980). In vitro effect of isoprinosine on rabies virus. Am. J. Vet. Res..

[32] Glasgow L.A., Galasso G.J. (1972). Isoprinosine: Lack of antiviral activity in experimental model infections. J. Infect. Dis..

[33] Skehan P., Storeng R., Scudiero D., Monks A., McMahon J., Vistica D., Warren J.T., Bokesch H., Kenney S., Boyd M.R. (1990). New colorimetric cytotoxicity assay for anticancer-drug screening. J. Natl. Cancer Inst..

[34] Ngoc N.T., Hanh T.T.H., Thanh N.V., Thao D.T., Cuong N.X., Nam N.H., Thung D.C., Kiem P.V., Minh C.V. (2017). Cytotoxic Steroids from the Vietnamese Soft Coral *Sinularia leptoclados*. Chem. Pharm. Bull..

[35] Roy S., Mukherjee S., Pawar S., Chowdhary A. (2016). Evaluation of In vitro Antiviral Activity of *Datura metel* Linn. Against Rabies Virus. Pharmacognosy Res..

[36] Klimstra W.B., Ryman K.D., Johnston R.E. (1998). Adaptation of Sindbis virus to BHK cells selects for use of heparan sulfate as an attachment receptor. J. Virol..

[37] Ramakrishnan M.A. (2016). Determination of 50% endpoint titer using a simple formula. World J. Virol..

[38] Wu Z.C., Wang X., Wei J.C., Li B.B., Shao D.H., Li Y.M., Liu K., Shi Y.Y., Zhou B., Qiu Y.F. (2015). Antiviral activity of doxycycline against vesicular stomatitis virus in vitro. FEMS Microbiol. Lett..

[39] Yang Q., Zhang Q., Tang J., Feng W.H. (2015). Lipid rafts both in cellular membrane and viral envelope are critical for PRRSV efficient infection. Virology.

[40] Luo Z., Tian D., Zhou M., Xiao W., Zhang Y., Li M., Sui B., Wang W., Guan H., Chen H. (2015). lambda-Carrageenan P32 Is a Potent Inhibitor of Rabies Virus Infection. PLoS ONE.

[41] Isaacs C.E., Wen G.Y., Xu W., Jia J.H., Rohan L., Corbo C., Di Maggio V., Jenkins E.C., Hillier S. (2008). Epigallocatechin gallate inactivates clinical isolates of herpes simplex virus. Antimicrob. Agents Chemother..

[42] Shi Z., Sun J., Guo H., Tu C. (2009). Genomic expression profiling of peripheral blood leukocytes of pigs infected with highly virulent classical swine fever virus strain Shimen. J. Gen. Virol..

[43] Sun J., Jiang Y., Shi Z., Yan Y., Guo H., He F., Tu C. (2008). Proteomic alteration of PK-15 cells after infection by classical swine fever virus. J. Proteome Res..

[44] Li L., Wang H., Jin H., Cao Z., Feng N., Zhao Y., Zheng X., Wang J., Li Q., Zhao G. (2016). Interferon-inducible GTPase: A novel viral response protein involved in rabies virus infection. Arch. Virol..

[45] Chavez J.H., Leal P.C., Yunes R.A., Nunes R.J., Barardi C.R., Pinto A.R., Simoes C.M., Zanetti C.R. (2006). Evaluation of antiviral activity of phenolic compounds and derivatives against rabies virus. Vet. Microbiol..

[46] Lee K.W., Hur H.J., Lee H.J., Lee C.Y. (2005). Antiproliferative effects of dietary phenolic substances and hydrogen peroxide. J. Agric. Food Chem..

[47] Hu H., Lee H.J., Jiang C., Zhang J., Wang L., Zhao Y., Xiang Q., Lee E.O., Kim S.H., Lu J. (2008). Penta-1,2,3,4,6-*O*-galloyl-β-d-glucose induces p53 and inhibits STAT3 in prostate cancer cells in vitro and suppresses prostate xenograft tumor growth in vivo. Mol. Cancer Ther..

[48] Kane C.J., Menna J.H., Sung C.C., Yeh Y.C. (1988). Methyl gallate, methyl-3,4,5-trihydoxybenzoate, is a potent and highly specific inhibitor of herpes simplex virus in vitro. II. Antiviral activity of methyl gallate and its derivatives. Biosci. Rep..

[49] Tillekeratne L.M., Sherette A., Fulmer J.A., Hupe L., Hupe D., Gabbara S., Peliska J.A., Hudson R.A. (2002). Differential inhibition of polymerase and strand-transfer activities of HIV-1 reverse transcriptase. Bioorg. Med. Chem. Lett..

[50] Serkedjieva J., Hay A.J. (1998). In vitro anti-influenza virus activity of a plant preparation from *Geranium sanguineum* L.. Antiviral Res..

[51] Campbell G.R., Bruckman R.S., Herns S.D., Joshi S., Durden D., Spector S.A. (2018). Induction of autophagy by PI3K/MTOR and PI3K/MTOR/BRD4 inhibitors suppresses HIV-1 replication. J. Biol. Chem..

[52] Li M., Li J., Yang J., Liu J., Zhang Z., Song X., Yao Z., Ma C., Li W., Zeng R. (2018). RSV replication is promoted by autophagy-mediated inhibition of apoptosis. J. Virol..

[53] Hu H., Chai Y., Wang L., Zhang J., Lee H.J., Kim S.H., Lu J. (2009). Pentagalloylglucose induces autophagy and caspase-independent programmed deaths in human PC-3 and mouse TRAMP-C2 prostate cancer cells. Mol. Cancer Ther..

[54] Dong Y., Yin S., Jiang C., Luo X., Guo X., Zhao C., Fan L., Meng Y., Lu J., Song X. (2014). Involvement of autophagy induction in penta-1,2,3,4,6-*O*-galloyl-β-d-glucose-induced senescence-like growth arrest in human cancer cells. Autophagy.

