# Supplementary material for: Inhibition of Rabies Virus by 1,2,3,4,6-Penta-O-galloyl-β-d-Glucose Involves mTOR-Dependent Autophagy
Source: Viruses. 2018 Apr 17;10(4):201. doi: 10.3390/v10040201 (PMC5923495; doi:10.3390/v10040201)
Supplement: Supplementary file 1 [file viruses-10-00201-s001.pdf]

Supplementary Information

Supplementary Figures:

Figure S1

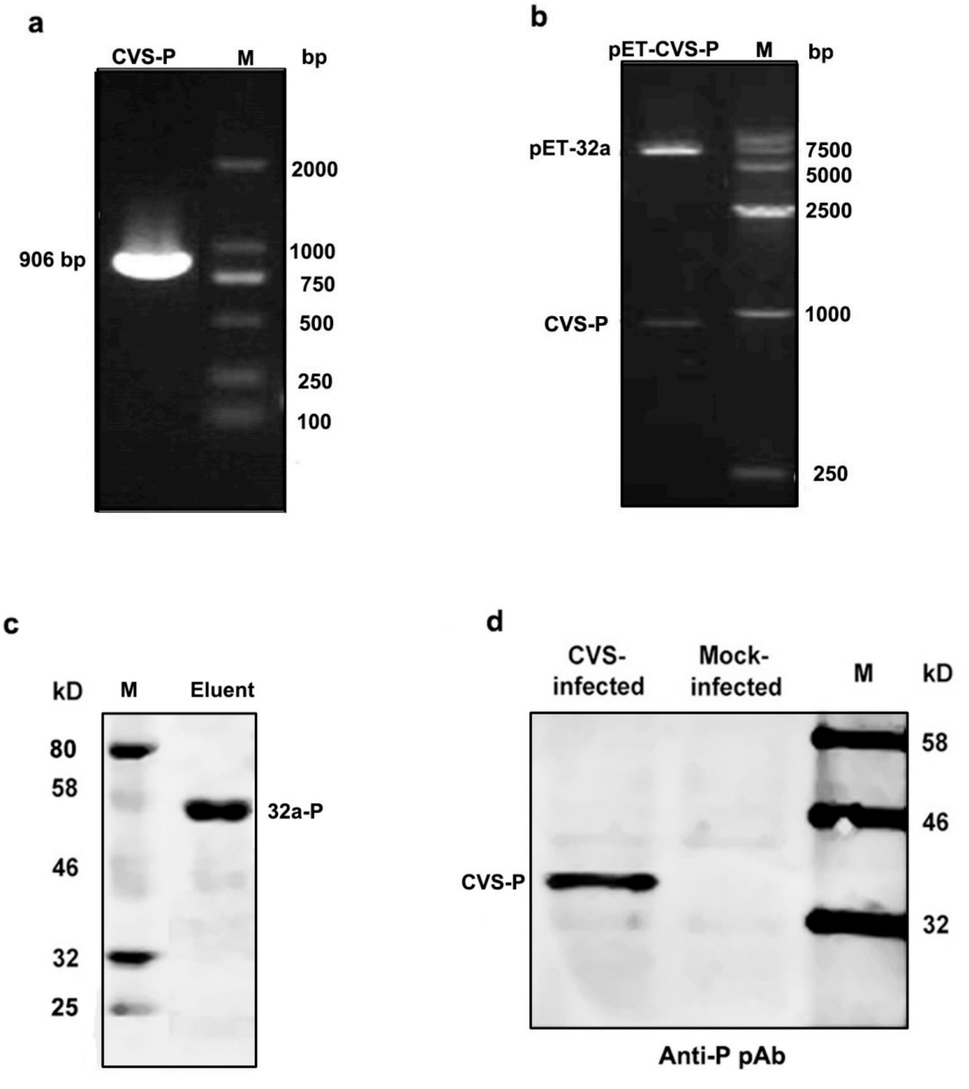

Figure S1. Preparation and identification of anti-RABV P protein polyclonal antibody (pAb). (**a**) Amplification of the RABV P gene by PCR. (**b**) Identification of recombinant plasmid by the restriction digestion with *EcoR*I and *Sal*I. (**c**) Identification of the purity of the purified recombinant protein 32a-P by SDS-PAGE. (**d**) Identification of the specificity of the polyclonal antibody by Western blotting.

Figure S2

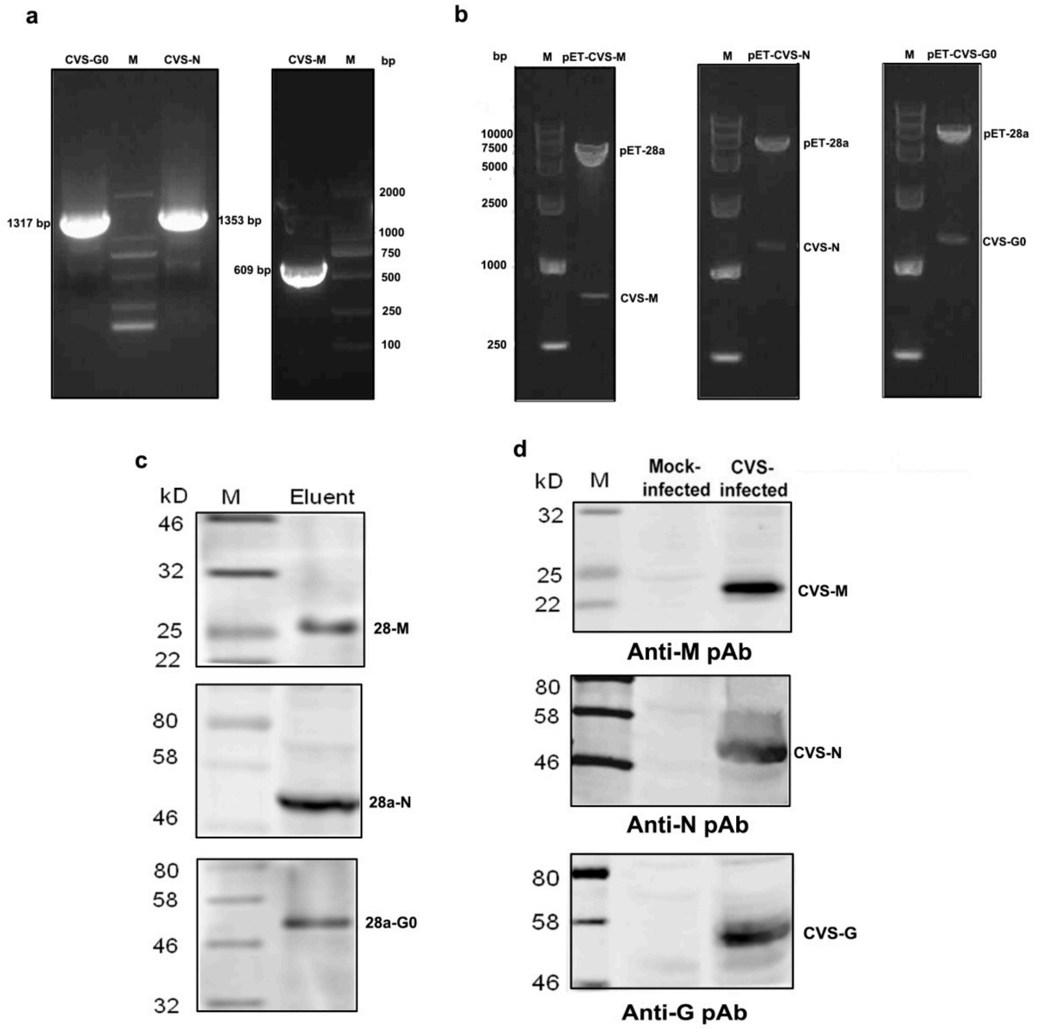

Figure S2. Preparation and identification of anti-RABV M, N, and G proteins polyclonal antibodies. (**a**) Amplification of the RABV M and N genes by PCR. (**b**) Identification of recombinant plasmid by restriction digestion. (**c**) Identification of the purity of purified recombinant protein by SDS-PAGE. (**d**) Identification of the specificity of polyclonal antibodies by Western blotting.

Supplemental Methods

Preparation and identification of polyclonal antibodies

The primers were designed to amplify the RABV M, N, G0 (extracelluar domain) genes from CVS-infected BHK-21 cells by PCR, for construction of the recombinant prokaryotic expression plasmid pET-28a-X. Recombinant plasmid was then identified by restriction digestion with *EcoR*I and *Sal*I. After induction and and purification of the recombination protein 28a-X with Ni-NTA His-bind resin (Merck Millipore), the purity was identified by SDS-PAGE. Finally, the purified recombination protein 32a-P (50 μg) was used to immunize the Kumming mice by multipoint subcutaneous injection in Freund's adjuvant, with a total of three times of immunization with a time interval of two weeks. Upon collecting the anti-X protein polyclonal serum, polyclonal antibody was purified by HiTrap Protein G HP (GE Healthcare Life Sciences) and identified by Western blotting.
